# “It's Not Like Taking Chocolates”: Factors Influencing the Feasibility and Sustainability of Universal Test and Treat in Correctional Health Systems in Zambia and South Africa

**DOI:** 10.9745/GHSP-D-19-00051

**Published:** 2019-06-24

**Authors:** Stephanie M. Topp, Candice M. Chetty-Makkan, Helene J. Smith, Lucy Chimoyi, Christopher J. Hoffmann, Katherine Fielding, Stewart E. Reid, Abraham J. Olivier, Harry Hausler, Michael E. Herce, Salome Charalambous

**Affiliations:** a College of Public Health, Medical and Veterinary Sciences, James Cook University, Queensland, Australia.; b Centre for Infectious Disease Research in Zambia (CIDRZ), Lusaka, Zambia.; c The Aurum Institute, Johannesburg, South Africa.; d Johns Hopkins University, Baltimore, MD, USA.; e London School of Hygiene & Tropical Medicine, London, UK.; f Division of Infectious Diseases, Department of Medicine, University of Alabama at Birmingham, School of Medicine, Birmingham, AL, USA.; g TB/HIV Care Association, Cape Town, South Africa.; h Institute for Global Health & Infectious Diseases, University of North Carolina School of Medicine, Chapel Hill, NC, USA.; i School of Public Health, University of Witwatersrand, Johannesburg, South Africa.

## Abstract

**Background:**

Sub-Saharan African correctional facilities concentrate large numbers of people who are living with HIV or at risk for HIV infection. Universal test and treat (UTT) is widely recognized as a promising approach to improve the health of individuals and a population health strategy to reduce new HIV infections. In this study, we explored the feasibility and sustainability of implementing UTT in correctional facilities in Zambia and South Africa.

**Methods:**

Nested within a UTT implementation research study, our qualitative evaluation of feasibility and sustainability used a case-comparison design based on data from 1 Zambian and 3 South African correctional facilities. Primary data from in-depth interviews with incarcerated individuals, correctional managers, health care providers, and policy makers were supplemented by public policy documents, study documentation, and implementation memos in both countries. Thematic analysis was informed by an empirically established conceptual framework for health system analysis.

**Results:**

Despite different institutional profiles, we were able to successfully introduce UTT in the South Africa and Zambian correctional facilities participating in the study. A supportive policy backdrop was important to UTT implementation and establishment in both countries. However, sustainability of UTT, defined as relevant government departments' capacity to independently plan, resource, and administer quality UTT, differed. South Africa's correctional facilities had existing systems to deliver and monitor chronic HIV care and treatment, forming a “scaffolding” for sustained UTT despite some human resources shortages and poorly integrated health information systems. Notwithstanding recent improvements, Zambia's correctional health system demonstrated insufficient material and technical capacity to independently deliver quality UTT. In the correctional facilities of both countries, inmate population dynamics and their impact on HIV-related stigma were important factors in UTT service uptake.

**Conclusion:**

Findings demonstrate the critical role of policy directives, health service delivery systems, adequate resourcing, and population dynamics on the feasibility and likely sustainability of UTT in corrections in Zambia and South Africa.

## Abstract (precis)

Universal test and treat may be feasible even in highly resource-constrained correctional facilities. Sustainability and impact of such services require a supportive policy environment, robust service delivery systems, adequate resourcing, and close attention to the psychosocial factors influencing incarcerated persons' willingness to engage in HIV treatment.

## BACKGROUND

Sub-Saharan African correctional facilities concentrate large numbers of people living with HIV and at risk for HIV infection. They include people from impoverished communities, those with poor baseline health, and those who engage in high-risk behaviors, such as commercial sex workers or individuals with substance use disorders.^1^^,^^2^ As a result, incarcerated populations in sub-Saharan African correctional facilities, who encompass both sentenced offenders and those detained and awaiting trial (on remand), experience higher HIV and tuberculosis (TB) prevalence than the general population.^3^

Studies from Zambia and South Africa, 2 countries among those having the largest correctional populations and generalized HIV epidemics in Southern Africa, have reported HIV prevalence among incarcerated persons between 12.5% and 27.4% (Zambia) and between 7.2% and 18.9% (South Africa), and TB prevalence of 0.34% to 7.6% (Zambia) and 0.71% to 3.6% (South Africa).^3^ The right to health obligates governments to provide health care to incarcerated people. To effectively prevent, treat, and care for HIV among incarcerated people, services must be tailored to their unique needs and HIV risk profile.^4^ Such efforts are necessary both because the demographic characteristics of and disease burden faced by incarcerated populations differ from those of the general population in the community and because the operational environment of correctional facilities are particular.^1^^,^^3^^,^^4^

Universal test and treat (UTT) is widely recognized as a promising approach to improve the health of individuals living with HIV and a population health strategy to reduce the incidence of new HIV infection.^5^ The benefits of early treatment have been demonstrated in various clinical trials documenting reduced risk of HIV transmission when viral load is suppressed, and reductions in HIV incidence with increasing population-level antiretroviral therapy (ART) coverage.^6^^–^^8^ Proponents of UTT have also highlighted the potential for a scaled-up approach to reduce the many barriers faced by people living with HIV to starting ART, and World Health Organization guidelines now recommend immediate ART initiation for all people living with HIV globally.^9^^,^^10^ Yet little is known about barriers to or facilitators of establishing and maintaining UTT in nontraditional programmatic settings such as correctional facilities.

With the aim of delivering a UTT intervention to incarcerated individuals in correctional health systems, we conducted the Treatment as Prevention (TasP) study in 3 correctional facilities in South Africa and 1 correctional facility in Zambia. Given the lack of evidence to date regarding UTT in correctional systems anywhere, a defined aim at the outset was to qualitatively explore the feasibility and compare the challenges to sustainability of this intervention within correctional facilities in 2 correctional systems.

We aimed to qualitatively explore the feasibility and sustainability of UTT within correctional facilities.

## METHODS

### Setting

Zambia has a network of 88 correctional facilities (some male only, some co-joined male/female) including several large maximum and medium security sites and a host of smaller district and farm prisons with a total official capacity of 6,100.^11^ Occupancy levels are high. In 2018, the Zambia prison population was estimated to be 25,000,^12^ representing a 34% increase since 2015 and an occupancy rate of 300%.^3^ The facility in which the current study was located is a medium-security facility in Zambia's capital city, Lusaka, and houses both convicted and “on-remand” individuals. It has a co-joined male and female wing, and at the time of study, it housed approximately 1,241 male and 129 female incarcerated persons, indicating an occupancy rate of over 300%.^11^ Administrative responsibility for corrections falls to the Zambia Correctional Service (ZCS) within the Ministry of Home Affairs.

South Africa has a network for some 243 correctional facilities ranging from maximum security to Community Corrections sites. South Africa's correctional system serves a population of over 158,000 incarcerated people.^12^ It has the 12th highest incarcerated population in the world, with an occupancy rate of 135%.^13^^,^^14^ Correctional facilities are administrated by a dedicated government ministry, the Department of Correctional Services. The South African sites in this study included 3 correctional facilities with 8 units. The Johannesburg Correctional Facility included 1 male maximum, 1 male medium, and 1 female unit, for a total population of 9,171. In Breede Valley, Branvlei Correctional Facility had 1 male maximum, 1 male medium, and 1 youth (ages 18–22) unit, and Worcester Correctional Facility had 1 female and 1 male maximum unit, for a combined total population of 4,173.

### Objectives

In this nested qualitative study, we aimed to (1) critically examine and compare feasibility of establishing UTT within the Zambian and South African correction health systems, and (2) draw out and compare aspects of both the intervention and the different correctional health system contexts likely to support or inhibit sustainability of UTT in the future.

### Conceptual Framework

This study was designed with the understanding that health systems are complex, meaning their function and performance are determined by the dynamic interactions between health system “hardware” and health system “software,” respectively.^15^ Drawing on the work of Sheikh and colleagues,^15^ we define health system hardware as the tangible, material components of a system, such as infrastructure, health workers, drugs, and commodities. Health system software comprises the interests, values, relationships, and practice-based norms of the human stakeholders whose decisions and actions bring the health system to life.^15^ Critically, in health system analyses, recognition of the values, beliefs, and relationships of *service users* (clients and/or patients) is considered just as essential to understanding health system performance as those of providers, managers, and policy makers.^16^

Feasibility studies typically seek to answer the question “Can this [intervention] be done in a given setting or context?” Hardware and software factors commonly considered in relation to health service feasibility include the willingness of patients, providers, and health planners and managers to participate; the perceived appropriateness and convenience of the intervention; availability of appropriate resourcing; and logistical systems required to support the intervention.^17^

Sustainability is conceptually distinct from feasibility and, simply defined, may be thought of as the “capability of being maintained at a certain rate or level.”^18^ In the context of evaluating health programs such as UTT, sustainability may be considered^19^:

*the ability … to function effectively, for the foreseeable future, with high treatment coverage, integrated into available health care services, with strong community ownership, using resources mobilized by the community and government.*

Mirroring the inclusive understanding of health systems described above, evaluation of sustainability involves attention to the broader organizational and systems dynamics, not just the obvious issues of proximate resourcing or training considerations.^20^^,^^21^ Interactions between stakeholders, institutions, and beneficiaries (“relational” components) are particularly important in ensuring continuation of a program, including via planned or spontaneous adaptations.^18^^,^^22^^,^^23^ Our understanding of sustainability was informed by these considerations as well as Schell and colleagues' domains of sustainability, which include strategic planning, organizational capacity, program adaptation, program evaluation, and communication, as well as political support, funding stability, partnerships, and public health impacts.^24^

### Primary Data Collection

Nested within the intervention study, our qualitative evaluation of UTT feasibility and sustainability used a case-comparison design^25^ that considered Zambia's and South Africa's correctional health systems as distinct cases. Within each case, we sought primary data from in-depth interviews with male and female incarcerated respondents with and without HIV and corrections staff and policy makers. We conducted interviews with all respondent types at each site. The rationale for interviewing respondents without HIV who were not directly involved in the UTT intervention was to ensure data on the nature of correctional life and correctional health services more generally and obtain reflections on any impact UTT may have had on those services.

We held in-depth interviews with incarcerated respondents, corrections staff, and policy makers.

Recruitment for all interviews with incarcerated individuals was a mixture of purposive and opportunistic, targeting male and females with and without HIV at each site. Respondents with HIV were identified through the intervention study (i.e., during enrollment or treatment procedures); respondents without HIV were identified with the assistance of correctional health staff, but no participants were selected by correctional staff. All individuals were invited to participate by a member of the study team during private visits to the clinic.

Recruitment of correctional staff (both health and non-health officials) was a mixture of purposive and opportunistic, and it was based on the study team's progressive identification of individuals involved in some aspect of health service delivery or planning within each correctional facility. Participation of any staff member typically had to be cleared through the facility in-charge, and recruitment was subsequently carried out via private invitation, in person, by a member of the study team. Selection of senior administrators and policy makers was wholly purposive and based on the investigators' prior (and emerging) knowledge of their involvement and expertise in the study area. Recruitment was achieved via phone invitation, email, or occasionally in person.

All interviews were conducted in person by trained research assistants recruited separately in each country. All but one interviewer (in South Africa) had previous experience conducting qualitative research, and all interviewers received a 5-day training on interview guides and human subjects protection in corrections. Interviews ranged from 20 to 100 minutes, and all were audio-recorded with written consent from the participants. Recordings were translated into English (as necessary) and transcribed in one step. Research assistants fluent in the language of the interview compared the transcript to the audio-recording to assess accuracy, completeness, and compliance with formatting requirements. Any anomalies were addressed by the interviewer or supervisor.

The Table summarizes the interviews conducted in Zambia (13 incarcerated individuals, 8 providers and administrators) and South Africa (37 incarcerated individuals, 13 providers and administrators). Interviews were guided by a common question guide developed for each of the 3 categories of respondents (incarcerated individuals with and without HIV, health care providers, corrections officials and policy makers). For each category of respondent, a common English question guide was developed during a multiphase consultation with Zambian and South African team members, with careful consideration given to cross-site differences (e.g., site-specific probes to capture local cultural and administrative context). The guide for incarcerated individuals focused on experiences with project (TasP) services as relevant and other experiences with the correctional health services, probing to understand matters of access, provider attitude, responsiveness, and overall acceptability. Health worker and correctional staff guides were framed by enquiries about the planning, communication, adaptation, resourcing, and perceived impact of UTT alongside questions regarding the organizational capacity and the day-to-day decisions faced by health and correctional personnel. Interviews with administrators and policy makers focused on higher-level concerns including the alignment of UTT with existing policy and programmatic priorities at the national and departmental/ministry levels, and the role and sources of funding and interinstitutional relationships. The English guide for the incarcerated respondents was translated into other national languages in Zambia (Nyanja, Bemba, and Tonga) and South Africa (Afrikaans, isiZulu, isiXhosa, Sesotho, and Setswana), back-translated, and pilot tested by the in-country teams. Health worker and policy maker interviews in both countries were conducted exclusively in English.

**TABLE.** Interviews Conducted, by Site and Respondent Type

Site	Male Inmates	Female Inmates	Health Workers, Managers, Policy Makers	Total
Zambia	11	2	8	**21**
South Africa, Johannesburg	12	5	3	**20**
South Africa, Cape Town	20	0	10	**30**
**Total**	**43**	**7**	**21**	**71**

### Document and Program Data

In order to contextualize and triangulate our interview data, we additionally drew on publicly available national policy documents, study documentation, and investigator experience (e.g. implementation memos) to cross-reference and strengthen understanding and interpretation.

### Analysis

All transcripts were transcribed and translated (as necessary) in a single step into English by an experienced, bilingual research assistant. At both sites, transcripts were subsequently quality checked by a second bilingual investigator against the original recording.

Following close reading of a selection of the English transcripts, 3 authors (ST, HS, CC) consulted and co-developed a coding framework based initially on deductive reasoning (informed by Sheikh and colleagues' framework^15^ and drawing on domains of feasibility and sustainability identified in the literature^18^^,^^22^^–^^24^) and refined inductively during the first several rounds of reading and coding. Transcripts were imported into Nvivo QSR (V.10 Australia) for coding and theming. For each site, ST plus a site-specific author co-coded several transcripts to validate and refine the codes and then coded 5 transcripts each to ensure consistency with high intercoder agreement. All remaining transcripts from Zambia and South Africa, respectively, were then coded independently.

## RESULTS

The qualitative findings of this study are presented in 2 sections. Built from interview data and investigator memos, Box 1 (Zambia) and Box 2 (South Africa) outline the living conditions and health service access of incarcerated respondents as critical context. We subsequently present findings relating to the feasibility and sustainability of UTT in each country. Cross-country differences and learnings from these findings are drawn out further in the discussion section. We use the nomenclature “correctional facility” (rather than prison) and “incarcerated individual” (rather than prisoner or inmate) except in cases of a direct quotation.

BOX 1 **Living Conditions and Health Service Access in the Zambian Facility**

**General Conditions**

- Poor living conditions in most correctional facilities in the country, including the study site.
- Extreme overcrowding and lack of appropriate sleeping quarters.
- Male interviewees noted that a substantial proportion of male incarcerated persons lived in cells so crowded that they had to sleep in a seated, upright position on the floor due to lack of space and bedding.
- Most cells did not have an internal bathroom.
- Personal hygiene was hard to maintain due to the limited hours with access to running water or bathing trenches, and the high demand for the same.
- Female quarters were generally acknowledged to be less crowded, but still poor.
- Personal hygiene was reported as poor, and resources for cleaning products lacking.
- In both men and women's facilities, incarcerated people described persistent concerns about communicable diseases, particularly tuberculosis.
- Poor food provided by Corrections—inadequate quantities and very poor quality—were a ubiquitous complaint. Most incarcerated people noted that without support from family or friends, they would not have food adequate to meet their nutritional needs.

**Health Service Access**

- Health service access required seeking permission from “senior inmates” and officers.
- During the day, male incarcerated individuals had to first report to their cell captains (those with delegated authority from officers) and ask to be placed on the official “sick list,” which gave them permission to go to the clinic. Once at the clinic, however, access to services was not guaranteed because opening hours were limited due to limited health workers (typically 1 clinical officer and/or nurse per day) being available and gatekeeping by peer educators (appointed incarcerated individuals) who managed clinic queues.
- At night, access to health care was more precarious, requiring the cell captain to bang on the door to attract the attention of the night guard, who then had to be convinced to contact a health worker.
- Incarcerated study participants noted that the attitudes and responsiveness varied substantially between health workers and security personnel. They also noted that access was particularly difficult in cases in which there was a lack of external (observable) symptoms.

BOX 2 **Living Conditions and Health Service Access in the South African Facilities**

**General Conditions**

- Living conditions were described as adequate, despite some overcrowding.
- Most of the incarcerated people had their own bed, rotating between top and bottom bunks in cells with 10–40 individuals.
- Each cell had an internally accessible bathroom.
- Some cells were designated “smoking” or “nonsmoking” cells, although not all individuals had a choice in their allocation. Smoking inside was allowed, and lack of ventilation was an ongoing problem for nonsmokers.
- Overcrowding was most problematic in relation to sanitation and hygiene. Queues to use toilets and showers, which were often broken or blocked despite regular cleaning and maintenance, was described by respondents at all 3 sites.
- Insufficient cleaning products or watered-down cleaning products were reported to make it difficult to keep facilities clean. Since access to showers was limited, people bathed from buckets in some cases.
- Incarcerated individuals were provided with basic toiletries (toothbrush/toothpaste) by correctional services, but they relied on family or friends for other personal items.
- Female incarcerated individuals reported that provision for maintaining menstrual hygiene was poor.
- Mixed reports were given regarding food but no identifiable pattern was present across the sites. Some felt food was basic but sufficient, while others reported the portions were too small and lacked nutritional value.
- In all study facilities, food was available for purchase from an internal corrections shop.
- Incarcerated individuals with HIV were provided “special diets” by the correctional service in recognition of special needs.

**Health Care Access**

- Different wings or “mediums” at each facility are allocated specific days to access the corrections clinic where treatment for all types of illnesses were addressed at the same time. Cape Town had a “tellie parade” and Gauteng a “sick parade” in which individuals reported any medical complaints or requested medical care and queued for treatment at the corrections clinic overseen by a nursing sister.
- Incarcerated individuals required an accompanying correctional official to access the corrections health clinic; this process was problematic due to staff shortages.
- Chronic medication such as antiretrovirals were provided weekly, and incarcerated individuals were allocated a specific day to collect their treatment. Side effects to medication were not addressed immediately, and study participants described how these could only be reported and addressed by health workers on allocated days.
- Access to the corrections health clinic on the weekend was only provided to specific individuals with known preexisting conditions. Incarcerated individuals and health workers at both sites noted that access to care was often difficult due to the high demand, the limited days allocated for each medium, the sometimes limited number of health workers, as well as the attitudes and behaviors of individual correctional staff and health workers.
- In cases of emergency, incarcerated individuals were able to request to go to the clinic or hospital.
- Relationships with correctional staff or “members” was described as generally positive.
- However, male and female respondents consistently described high rates of emotional violence and fear of (more occasional) physical violence from other incarcerated persons. Interviewees painted an overall picture of basic but passable physical living conditions, but an extremely high-stress environment due to prevalent personal anxiety and tense interpersonal relationships among those incarcerated.

### Zambia

#### Feasibility of UTT in Zambia

UTT services in the Zambian correctional facility were implemented as a predominantly parallel service, with components that were physically distinct from the correctional facility's day-to-day health service. The decision to deliver UTT in this way was based on the project team's a priori knowledge of and pragmatic considerations relating to the constrained human and material resources available for health care within the Zambian correctional system.^11^ Standalone baseline clinical and laboratory evaluation and ART initiation services were delivered within the facility's (internal) clinic, but they were staffed and resourced exclusively by the study. Where possible, some service components were integrated within existing correctional health processes. For example, HIV testing for the study was largely driven by counselors and peer educators who worked as part of the correctional health service for HIV testing and counseling. Overall, implementation of UTT in this manner proved highly feasible at the study site (a large, centrally located urban study facility), with interview data and investigator experience pointing to 3 major contributing factors as described below.

UTT implementation proved highly feasible at the Zambian study site, with 3 major factors contributing to success.

#### Alignment With National Policy

Interviews with senior correctional and Ministry of Health (MOH) officials demonstrated a high level of support for the idea of establishing and scaling up UTT to all Zambian correctional facilities. Such support was set against Zambia's national policy of test and treat for HIV, introduced in 2017, which mirrors widespread political and bureaucratic enthusiasm for improved access to HIV care and treatment. One senior ZCS official noted that the policy meant that the opportunity to trial UTT in all correctional facilities was seen as an important development and was in line with “mainstream health services.”

#### “Open-Door” Policy and Long-Term Partnerships

The Zambian Correctional Service has an open-door policy, working with trusted partners to strengthen facility-based health services. This policy operates in recognition of profound resourcing and capacity limitations within ZCS and enables correctional officers to work with nongovernment groups to address the most urgent health resourcing and health service gaps. Since 2009 when the unofficial policy was put in place, relationships with several long-standing partners have evolved.

*The correctional service has opened its doors, to the stakeholders, to help provide health service, provide technical skills, provide funding, and this is helping to reorganize I think, the system for health delivery in the system. [ZCS official]*

The existing partnership between the Zambian implementing NGO and ZCS was an important facilitator in the establishment of UTT in the Zambian site. The relationship and prior experiences underpinned both initial planning and the permissions provided by the ZCS Commissioner General and the subsequent routine access afforded to study staff by the facility manager. This relationship was a vital enabler. Ad hoc challenges and miscommunications between study and correctional staff, an anticipatable challenge in any project, were substantially easier to manage in the context of the established relationship between the NGO and ZCS.

#### Positive Attitudes Toward HIV Care and Treatment

A final component of the feasibility of UTT in Zambia was the acceptable nature and comparatively strong demand for HIV testing and treatment services among the incarcerated population. Incarcerated individuals, health care providers, and ZCS officers all described the environment of the Zambian correctional facility as one that was generally open about and supportive of individuals seeking care. Several participants described how the encouragement they received to do an HIV test and look after their health when they first entered corrections were instrumental in their decisions to seek care.

*Even HIV, I did not even want to test when I was outside prison; because if I was outside prison maybe I was going to be saying I was bewitched. So I appreciate a lot because my coming to prison helped me to even test. [Female inmate, Zambia]*

Demand for health services in the study facility was in part underpinned by a comparatively low-stigma environment and a notable culture of peer support in relation to HIV testing and treatment uptake.

*At the moment, stigma is no longer there, we just live as friends because everyone inside has a group. But there is no choosing like this one is taking ARVs or is on TB [treatment]. [Male with HIV, Lusaka]*

Several respondents contrasted the comparatively lower HIV-related stigma in the men's facility with the still prevalent concerns about stigma in the mainstream (noncorrections) community. Longer-term incarcerated respondents and corrections staff attributed this difference to a series of rolling education and sensitization programs carried out by various nongovernment groups over several years leading up to UTT. Such an environment was instrumental in both the strong early uptake and sustained demand for testing and treatment services delivered in the male section of the Zambian site of this project. Notably, however, female respondents in the much smaller female section described more concerns regarding HIV-related gossip and stigma.

*Sometimes you find an old [timer] inmate maybe stigmatizing another one. Mmhh, it was terrible, it was terrible. When your friend is very sick, looking at the congestion, you know we have to sleep like bumper to bumper. People were scared to sleep next to a sick person, thinking they will also catch that same disease. [Female without HIV, Lusaka]*

#### Sustainability of UTT in Zambian Correctional Facilities

While the data point to the feasibility of implementing UTT as a largely vertical, NGO-supported service, we found more equivocal evidence in relation to the long-term sustainability of UTT, at least in terms of a government (ZCS and MOH)-led program. Mirroring some known issues, we identified the follow factors undermining the sustainability of the UTT approach: weak ZCS funding and resourcing for health activities; still limited organizational capacity of the Zambian correctional health system; and a policy backdrop that while supportive, did not make the need for UTT in correctional facilities sufficiently explicit. Reflecting both deductive and inductive themes, we identified barriers and facilitators relating to 3 major sustainability domains as outlined below.

We found equivocal evidence regarding the long-term sustainability of UTT at the Zambian facility.

**Policy Backdrop:** Zambia's national policy for UTT introduced in 2017 helped to establish a backdrop of robust political support. However, ZCS and MOH stakeholders flagged the lack of *explicit* mention of correctional facilities or incarcerated populations as part of UTT scale-up as a potential problem. Lack of explicit mention of correctional services raised questions about whether national (MOH-controlled) resourcing for and support of UTT scale-up would cover the introduction and maintenance of UTT in correctional facilities, as an MOH official described:

*So the universal testing, counseling, and treatment for Zambia, it refers to the health facilities both public and private. [And although] that can be broadly translated to the prison service [at the moment] technically it does not apply; it does not apply to non-health settings. Which is a challenge. [MOH official]*

In the context of the lack of depth in the health service leadership of ZCS, moreover, the absence of explicit guidelines to support the scale-up of UTT services was described as a considerable barrier.

**Funding and Resourcing:** The potentially narrow interpretation of “universal” test and treat as only applying to mainstream health services was a significant issue from both a resourcing and organizational standpoint. During the study, implementation of UTT in the Zambian correctional facility was dependent on external (study) funding. At the time of writing, additional external resources from 3 different donor-funded programs had been found to support continued provision of UTT in the short to mid term. But few immediate prospects for additional internal resources to maintain the staff or systems exist. Various stakeholders described a range of related resourcing concerns relating to the sustainability of UTT within the correctional service. These most notably included inadequate numbers and inappropriate skill mix of current human resources for health and largely inadequate health infrastructure that mitigated against the delivery of high-quality care.

*You find that inmates will be many and there will only be one nurse. And then they will just say: “These 15 will be seen, the other ones will be seen in the afternoon.” But maybe they don't attend to even those. [Male with HIV, Lusaka]*

*The infrastructure in correctional services for health … they are all dilapidated. And so we do not have an appropriate system where the services can be provided in a quality manner … . [ZCS official]*

The potentially narrow interpretation of UTT was a significant issue from both resourcing and organizational standpoints.

**Organizational Capacity and Organizational Culture:** Related to, but distinct from lack of resourcing as a barrier to sustained UTT, was the lack of depth and capacity for planning or delivery of care. While the aforementioned absence of human resource capacity and a dedicated health budget underpin these problems, lack of depth and capacity in leadership and supervisory roles also contributed to poorly constituted and weakly integrated health information systems and weak supply chains.

*The other weakness is in terms of reporting systems. We have developed [paper-based] tools … but if we had computers, emails, so that we are able to monitor what is happening, in a second you are able to see what is happening in [X facility]. But we normally receive hard copies, when they send it from [X facility] to here. It may take 2 weeks to a month to reach here. [ZCS official]*

Despite efforts to ameliorate such issues, lack of depth in health leadership also contributed to an often unresponsive health service culture, constituting an additional and distinct barrier to sustained provision of UTT in this setting. Various incarcerated respondents and a corrections-based health worker commented on the way these issues affected both HIV and more generalized health service access and uptake:

*[In the Corrections clinic] you find that today the clinical officer is not there, today the nurse is not there. [Male with HIV, Lusaka]*

*You know they don't treat us as patients. [Corrections health providers] treat us as criminals […] so even if you come for treatment when you are sick, they are harsh on you. [Male with HIV, Lusaka]*

### South Africa

#### Feasibility of UTT in South Africa

Implementation of UTT in the 3 South African correctional facilities was negotiated between local project teams and Department of Correctional Services (DCS) staff separately at each of the 3 sites. In Johannesburg, the project team only conducted ART initiations; HIV testing was conducted by another NGO team as part of a Global Fund–supported service. In the 2 Western Cape sites, the project team ensured that ART medications were available in all instances, but UTT services (testing and treatment initiation) were delivered by the DCS clinic team as part of routine care. In all sites, incarcerated respondents' and DCS stakeholders' accounts demonstrated the feasibility of delivering UTT within these particular South African correctional facilities, with interview data and investigator experience pointing to 2 major contributing factors as described below.

The feasibility of delivering UTT within South African correctional facilities drew on 2 major contributing factors.

#### National UTT Policy and Study Resourcing

Prior to project start on September 1, 2016, the South African National Department of Health (NDOH) introduced a national UTT policy, which provided the criteria for starting all patients with HIV on lifelong ART. Since DOH guidelines for the management of HIV apply to correctional centers^3^ in South Africa, this change in national policy was widely understood to apply to correctional settings. Interviews highlighted how knowledge of this policy among both frontline and administrative DCS staff facilitated the establishment of UTT in correctional facilities. Despite some early fears among some to the contrary, DCS staff at all 3 sites additionally described how the study helped them absorb the backlog of counseling and paperwork associated with initiating many now-eligible individuals with HIV into HIV treatment.

*UTT, when it was initiated by the Department of Health, it became something very crucial for us [in corrections] But now, there's no backlog anymore [… the study made] it easier for us to get those inmates on treatment as soon as they were being diagnosed. [Nursing sister, South Africa]*

Study resources, which included personnel to help with HIV testing and counseling and importantly antiretroviral medications for newly initiated individuals at the study sites, as well as the development of tailored protocols, helped DCS health workers make the transition to UTT in the 2 study sites with relatively few disruptions to existing duties.

#### Correctional Health System and Established Partnerships

Respondents' descriptions of the health services in the 3 South African sites, combined with the investigators' experience, point to robust systems for the delivery of health care in the 2 South African correctional facilities. In particular, transparent processes for accessing HIV testing and treatment provided a platform from which UTT procedures could evolve. As a frontline provider noted:

*Prior to this project being implemented there was a certain working procedure towards HIV/AIDS and TB treatment, care and support [developed with our partners]. I think over the years we have, the wheels were welled oiled. [Nursing sister, South Africa]*

Reflecting the comparatively strong internal capacity of the South African correctional system, several DCS staff clearly positioned themselves as champions of UTT, serving as informal and formal conduits of information and advocates for necessary changes or adaptations to other DCS staff. Preexisting relationships between the (separate) NGO implementing partners at the 2 study sites and DCS officials also provided an important basis for communication and problem solving, helping overcome early challenges and misunderstandings.

#### Sustainability of UTT in South African Correctional Facilities

The prospects for sustained UTT in the 3 South African correctional facilities appeared strong, although barriers remained. Reflecting both deductive and inductive themes, we identified barriers and facilitators relating to 4 major sustainability domains as outlined below.

The prospects for sustained UTT in the South African facilities appeared strong, although barriers remained.

**Policy Backdrop and Political Support:** As described above, South Africa's national UTT policy was key to promoting acceptance of the TasP project. But as noted in the quote below, the policy also ensured that DCS staff were interested in facilitating and adopting UTT in the long term.

*Like I said with regards to UTT when it was initiated by the Department of Health it became something very crucial for us [to implement]. [DCS officer]*

This understanding that the impetus for UTT came from central government, rather than from the study itself, also contributed to buy-in by mid- and senior-level DCS officials in relation to the longer-term adoption of UTT. As one noted:

*[The study] helped us a lot [to] see how this is actually feasible. But even if [study funding] stops I think we must continue with [the] way we are working now. [Correctional HIV AIDS Coordinator, South Africa]*

However, some barriers to UTT sustainability were noted. Several health care providers described current DCS policies that do not empower nurse prescribing and ART initiation as a potential impediment to sustained and effective UTT, since ongoing shortages of medical doctors in the correctional system could lead to bottlenecks in treatment initiation.

**Funding and Resourcing:** Notwithstanding the relatively well-established systems in the South African correctional health services, a common theme was the “brittleness” of these services. Data revealed that corrections health services operated on a skeleton staff who had little time to deal with anything beyond routine check-ups. Frontline health workers at all 3 sites described chronic (reportedly system-wide) staff shortages that necessitated a daily juggling act as they tried to deliver services for both acute and chronic conditions across a large population with multiple, often complex physical and mental health care needs.

*Nurses are under staffed again and over worked because it's a lot of [health] complaints. … I think that [shortage] can have a very negative effect on the health care service delivery. [Correctional HIV AIDS Coordinator, South Africa]*

The implications for understaffing for UTT were many, but one standout consequence emerging from interview data was the inability of DCS to deliver on critically important psychosocial counseling during initial HIV testing or as part of ongoing HIV treatment. DCS-employed HIV counselors or other psychosocial supporters were limited. Yet their services were described independently by both incarcerated individuals and providers as essential to incarcerated individuals' uptake of and sustained engagement in HIV care and treatment. Favorable comparisons were made by respondents at the various sites to the counseling made available during the study, when TasP staff with dedicated counseling roles bolstered health worker numbers:

*It's not like taking chocolates and everything, you know. You've got to be, ja [hand gesture]. And then we received such perfect, perfect counseling with [the TasP team]. And it makes it so easy for us, you know, to, to take our pills each and every day. Because we know the purpose, we know why we are taking these pills. Ja man, prison! [Male inmate, Cape Town]*

**Organizational Capacity and Organizational Culture:** The comparatively well-established health services within DCS were facilitators of scaled-up and sustained UTT across the 3 South African correctional sites. Nonetheless, 2 corrections-specific organizational barriers were identified. The first, related to health information challenges. These included still weak internal health information systems and resulting communication breakdowns during transfers of patients between corrections facilities. Inconsistent communication between security and health personnel and nonharmonized health information systems between facilities were described as often resulting in missing or delayed medical records that left patients on ART without access to medication. At a higher level, lack of harmonization between DCS and DOH indicators for HIV care and treatment were identified as likely inhibiting long-term monitoring.

The second aspect of organizational capacity related to the weak health literacy of security personnel and its impact on service organization and access. As described by one security officer:

*We did not receive training on the college to work with [NGO] personnel or with providing of treatment [to a] person. We did not get that kind of training at Corrections College. Uhm, that is why some of the [staff] felt that: “it's not my job description. Where do these people come from now?” [Security officer, South Africa]*

Coordination between DCS security and health personnel were described as challenging in some situations, potentially limiting incarcerated peoples' access to care. Efforts to ensure leadership and communication between different categories of corrections staff were described as an area requiring improvement to facilitate sustained and effective UTT over time.

**Population Dynamics and Service Demand:** A final theme relating to UTT sustainability in the South Africa corrections health system relates to population dynamics of each facility and their effect on UTT service demand. Incarcerated individuals, health workers, and DCS officials all described the correctional facilities as high-stress environments in which peer relations among the incarcerated were fraught and emotional violence was common.

*Some of our clients … suffer a lot with, for instance, depression. Because you know the prison system, the correctional system, is not a place where someone wants to be … their freedom is being taken from them, their families, their loved ones, their children can't see them every day. … And now you come and stay with total strangers, in a strange place … the medical or the psychological effect that it has on the inmates is something that we can't deny. [Nursing sister, Gauteng]*

Many incarcerated respondents and health providers described how, in this stressful and isolating environment, self and perceived stigma around HIV status was a barrier to accessing or remaining engaged in HIV treatment. HIV-related stigma was a prominent concern among the majority of the individuals with HIV who were interviewed, and it was confirmed by health care providers. Self-stigma, typically expressed as a reluctance to reveal or disclose to anyone else, was also frequently described, exacerbated by poor HIV treatment literacy, lack of personal coping mechanisms, and myths and misconceptions around the effects and implications of starting ART.

*It is a challenge, it is a challenge, because at first when [stuttering] you are told that you're actually now having to live with the fact you are HIV, you know, your mind shuts [down] immediately. You know, you [stuttering] get confused and you think of [stuttering], the environment now that you are in. I think it would be much better if I was outside, but now I'm in prison, you know, where we are ten inmates in a small [single] cell, and these people will see that I'm taking pills. [Male inmate, Gauteng]*

Individually and in combination, these factors were consistently linked by different types of respondents to poor uptake of existing (pre-UTT) HIV testing and to poor adherence to ART.

*We are not on 100% compliance with regards to ARV treatment even now. Not because it's not available … Some of them said [they don't want to start treatment] because of their family. Their culture says they mustn't. Others say they just don't want it. But if you go and dig a bit deeper, you can see that …] they don't want to do because they will be labeled and stigmatized and all those kind of things. So, they trying to keep their status confidential by not taking their medication because the moment they start taking their medication, there is no confidentiality. [Nursing sister, Cape Town]*

Some women who had chosen not to start ART, raised concerns regarding the “double sentence”—physical imprisonment and the “sentence” of HIV. These individuals explained that they did not want to have to worry about HIV or initiate treatment until they were released. Against this backdrop, the weak presence of HIV-specific or more generalized psychosocial counseling or support services in the South African correction health services was described as a substantial limitation to sustained and effective UTT.^26^

## DISCUSSION

Recent expansion of HIV testing and treatment in heavily HIV-burdened countries has been framed as a global imperative. However, the key populations designated by Joint United Nations Program on HIV and AIDS as being at greatest risk for HIV, including incarcerated peoples, are being left behind in the global response, often going without proper access to HIV treatment and other health services.^27^ While large-scale clinical trials have demonstrated the effectiveness of new biomedical technologies for tackling HIV, a number of unanswered questions remain, particularly with regard to *how* we implement these technologies for certain populations and in various subnational contexts.^28^ Indeed, information about or evidence from interventions to help address HIV care and treatment among incarcerated populations in sub-Saharan Africa and elsewhere, remains limited.

To our knowledge, and notwithstanding the overwhelming burden of HIV in sub-Saharan Africa, this study is the first to qualitatively explore the feasibility and sustainability of UTT in any African correctional system. A key contribution of this study is its consideration of both the interventional *and* contextual factors likely to affect UTT, synthesized through comparison of the influence of the different political, correctional, and health service delivery systems in Zambia and South Africa, respectively. It is also one of still very few articles^29^^–^^31^ from the region describing any form of health intervention in the correctional context. Given the dearth of literature, we focus the Discussion on 3 major, cross-cutting lessons synthesized from the findings presented above, which are relevant to the broader study questions but also important flags for policy makers and programmers who may be considering UTT roll-out in correctional systems elsewhere.

A key contribution of this study is its consideration of the interventional *and* contextual factors likely to affect UTT.

### National Policies and Associated Resourcing Are Critical

Both the Zambian and South African experiences in this study demonstrated the critical influence of national HIV treatment policies on the feasibility and sustainability of UTT in *correctional* health systems, albeit in different ways. In South Africa, the national policy was interpreted as inclusive of incarcerated populations, influencing the response of DCS officials and facility-level providers who felt compelled to implement UTT in correctional facilities. In Zambia, the national UTT policy ensured general support for the project. However, the lack of clarity at a high level about whether the policy—a form of clinical guidance—guaranteed the necessary resources had implications not only for the capacity of ZCS to implement it, but also for correctional officials' sense of urgency or compunction to comply with it. These experiences highlight the importance of understanding both the political and resourcing context of policy change, and they mirror concerns elsewhere about the lack of specificity in nominally universal health policies, which can falter in operationalization due to differing interpretations of scope, intent, or measurement.^32^

National HIV treatment policies strongly influence the feasibility and sustainability of UTT in correctional health systems.

Although less apparent in our primary data, author experience and a critical body of literature on correctional health in sub-Saharan Africa^4^^,^^11^^,^^14^^,^^33^ point to the key role that reform of the justice system (e.g., bail alternatives to limit over-incarceration) could play in making HIV prevention and treatment more effective and affordable. Such reforms are central to tackling upstream drivers of incarcerated people's lack of access to care (e.g., by reducing overcrowding through parole reform) and constitute a fundamental and complementary set of strategies in the quest for sustained deliver of UTT.

### Corrections Health Delivery Systems and Organizational Capacity

The influence that the capacity of the correctional health system had on the feasibility and sustainability of UTT in correctional facilities is an intuitive but central finding. In Zambia, some recent progress in establishing internal health systems was noted,^29^^,^^31^ but it was insufficient to independently support and sustain UTT. Chronic shortages of health providers on site, a still minimally staffed ZCS Health Directorate, a nearly absent health information system, and a weak supply chain undermined independent (ZCS-led) health service planning and delivery. With a strong recent history of partnership between ZCS and a range of nongovernmental implementing partners, however, a hybrid model of care based on public-NGO partner models is likely sustainable.^34^^,^^35^ Investment in the longer-term health system management and planning capacity building for frontline officers and mid- and upper-level ZCS officials nonetheless remains an important priority.^36^^,^^37^

South Africa's correctional health services showed capability to take on and adopt UTT. However, mirroring the experience of UTT implementation in mainstream South African health services,^38^ the study also revealed a number of issues regarding scalability and sustainability. Reflecting concerns in other recent literature on South Africa's correctional system,^39^^,^^40^ our findings demonstrated an urgent need to boost human resource capacity and ARV stocks to manage the surge of testing and treatment initiation created by the backlog of treatment-eligible incarcerated people. The study also revealed how current national clinical guidelines that require a medical doctor to initiate ART are already contributing to treatment bottlenecks in correctional facilities where doctors are few, a situation likely to be exacerbated by UTT. Limited provision of essential pre- and posttest counseling and psychosocial support for clients with HIV were also linked to gaps in skills mix of the correctional health workforce. Finally—similar to, although less acute than in Zambia—findings highlighted the need to strengthen correctional health information systems to enable better linkage and tracking between correctional facilities and across the corrections and mainstream health systems.

An urgent need exists to boost human resource capacity and ARV stocks to manage testing and treatment among incarcerated people.

### HIV Stigma and Service Demand

Reflecting the importance of health system software on the feasibility and sustainability of UTT in corrections, we found HIV stigma and associated service demand within correctional facilities to be a key cross-cutting theme. In the Zambian female section and in South African facilities, respondents described HIV-related stigma as an ongoing barrier to UTT uptake. Accounts referenced recent and past trauma that interacted with feelings of fear and isolation, leaving many individuals unwilling to start treatment even where they knew that testing and treatment were accessible.

Our findings align with previous research among incarcerated males in South Africa that has traced the psychosocial determinants of risk behavior^41^ and in which respondents' narratives of fear and isolation segued into descriptions of (emotional and psychological) barriers to health care access. Various (noncorrectional) studies have also traced the influence of psychological, social, and cultural factors on HIV care seeking and willingness to enroll in ART in other sub-Saharan African countries.^38^^,^^42^^–^^44^ This qualitative study, with its focus on correctional health systems, was not specifically designed to assess mental health, although a mental health assessment of enrollees with HIV in the clinical study was conducted and will be reported elsewhere. Nonetheless, the narratives of incarcerated individuals from both countries pointed to the likely intersectionalities between incarcerated people's mental health, stigma, and HIV. From a service and treatment efficacy perspective, it seems reasonable to assume that poor mental health among incarcerated people affects their agency to seek and remain engaged in care. Future work should more explicitly explore these links and their implications for the profile and the skills mix of health care services and providers in correctional settings.

The narratives of incarcerated individuals pointed to intersectionalities between mental health, stigma, and HIV.

Counterintuitively, interviews with incarcerated males in the Zambian facility in this study described a strong culture of peer support for HIV testing and treatment, describing it as an important factor in their willingness to seek care, even where physical access was limited. As recent work has suggested that key populations in Zambia continue to face barriers to service utilization in the mainstream health system,^29^^,^^31^^,^^45^ more work is needed to explore and trace the factors that may have enabled and strengthened this unusual and encouraging situation in the correctional facility.

### Limitations

This study analyzed data relating to the implementation of UTT in 1 Zambian facility and several South African facilities. While we endeavored to garner views representative of different levels of the health system—client, frontline health worker, managers, and policy makers—in order to improve analytic generalizability, we acknowledge that both Zambia and South Africa have multiple correctional facilities that differ in size, security level, infrastructure, and administrative dynamics from the study sites. Zambia and South Africa do not prevent noncitizens from accessing health services in corrections, so we did not interview participants to reflect on their ability to access UTT. However, we did not interview any confirmed noncitizen incarcerated persons in either country, and we acknowledge that their experience of correctional services and ability to access UTT within such settings may well be different from that of citizens. Because the larger study in which this qualitative work was nested focused on implementation of UTT, we also did not focus explicitly on other service or upstream gaps (e.g., mental health and/or criminal justice reform) future work in corrections should seek to include such gaps in these and similar settings. Further work to identify the synergies of such services or reform efforts for ensuring (among many outcomes) long-term sustainability of HIV-specific services will be important.

## CONCLUSION

Despite different institutional profiles, introduction of UTT was feasible in correctional health systems in both South Africa and Zambia. A supportive policy backdrop was important to UTT establishment in both countries. However, the prospects for long-term sustainability of UTT differed. South Africa's correctional facilities had existing systems to deliver and monitor chronic HIV care and treatment, forming a scaffolding for sustained UTT despite some human resources shortages and poorly integrated health information systems. Notwithstanding recent improvements, Zambia's correctional health system demonstrated insufficient material and technical capacity to independently deliver quality UTT. In the correctional facilities of both countries, population dynamics and their impact on HIV-related stigma were important factors in UTT service uptake.

## Notes

**Peer Reviewed**

**Funding:** This research was funded through a grant from the DFID and SIDA-funded Evidence for HIV Prevention in Southern Africa (EHPSA) program: Grant Number: MMM/EHPSA/AURUM/05150013

## References

[1] Rubenstein LS Amon JJ McLemore M. HIV, prisoners, and human rights. Lancet. 2016;388(10050):1202–1214. 10.1016/S0140-6736(16)30663-8. 27427457

[2] Dolan K Wirtz AL Moazen B. Global burden of HIV, viral hepatitis, and tuberculosis in prisoners and detainees. Lancet. 2016;388(10049):1089–1102. 10.1016/S0140-6736(16)30466-4. 27427453

[3] Telisinghe L Charalambous S Topp SM. HIV and tuberculosis in prisons in sub-Saharan Africa. Lancet. 2016;388(10050):1215–1227. 10.1016/S0140-6736(16)30578-5. 27427448 PMC6182190

[4] Herce ME Muyoyeta M Topp SM Henostroza G Reid SE. Coordinating the prevention, treatment, and care continuum for HIV-associated tuberculosis in prisons: a health systems strengthening approach. Curr Opin HIV AIDS. 2018;13(6):492–500. 30222608 10.1097/COH.0000000000000505 PMC7705648

[5] Hontelez JAC Lurie MN Bärnighausen T. Elimination of HIV in South Africa through expanded access to antiretroviral therapy: a model comparison study. PLoS Med. 2013;10(10):e1001534. 10.1371/journal.pmed.1001534. 24167449 PMC3805487

[6] Cohen MS Chen YQ McCauley M; HPTN 052 Study Team. Prevention of HIV-1 infection with early antiretroviral therapy. N Engl J Med. 2011;365(6):493–505. 10.1056/NEJMoa1105243. 21767103 PMC3200068

[7] Grinsztejn B Hosseinipour MC Ribaudo HJ.; HPTN 052-ACTG Study Team. Effects of early versus delayed initiation of antiretroviral treatment on clinical outcomes of HIV-1 infection: results from the phase 3 HPTN 052 randomised controlled trial. Lancet Infect Dis. 2014;14(4):281–290. 10.1016/S1473-3099(13)70692-3. 24602844 PMC4144040

[8] National Institutes of Health, US Department of Health and Human Services. Starting antiretroviral treatment early improves outcomes for HIV-infected individuals [news release]. https://www.nih.gov/news-events/news-releases/starting-antiretroviral-treatment-early-improves-outcomes-hiv-infected-individuals. Published May 27, 2015. Accessed April 28, 2019.

[9] Brown LB Havlir DV Ayieko J.; SEARCH Collaboration. High levels of retention in care with streamlined care and universal test and treat in East Africa. AIDS. 2016;30(18):2855–2864. 10.1097/QAD.0000000000001250. 27603290 PMC5332142

[10] World Health Organization (WHO). Guideline on When to Start Antiretroviral Therapy and on Pre-Exposure Prophylaxis for HIV. Geneva: WHO; 2015. https://apps.who.int/iris/bitstream/handle/10665/186275/9789241509565_eng.pdf. Accessed April 10, 2019. 26598776

[11] Topp SM Moonga CN Luo N. Mapping the Zambian prison health system: an analysis of key structural determinants. Glob Public Health. 2017;12(7):858–875. 10.1080/17441692.2016.1202298. 27388512

[12] Institute of Criminal Policy Research (ICPR). World Prison Brief. London, United Kingdom: ICPR; 2018. http://www.prisonstudies.org/. Updated April 2019. Accessed April 28, 2019.

[13] Republic of South Africa, Department of Correctional Services (DCS). Annual Report 2016-2017. Pretoria, South Africa: DCS; 2017.

[14] Keehn EN Nevin A. Health, human rights, and the transformation of punishment: South African litigation to address HIV and tuberculosis in prisons. Health Hum Rights. 2018;20(1):213–224. 30008564 PMC6039737

[15] Sheikh K Gilson L Agyepong IA Hanson K Ssengooba F Bennett S. Building the field of health policy and systems research: framing the questions. PLoS Med. 2011;8(8):e1001073. 10.1371/journal.pmed.1001073. 21857809 PMC3156683

[16] Sheikh K Ranson MK Gilson L. Explorations on people centredness in health systems. Health Policy Plan. 2014;29(suppl 2):ii1–ii5. 10.1093/heapol/czu082. 25274634 PMC4202918

[17] Proctor E Silmere H Raghavan R. Outcomes for implementation research: conceptual distinctions, measurement challenges, and research agenda. Adm Policy Ment Health. 2011;38(2):65–76. 10.1007/s10488-010-0319-7. 20957426 PMC3068522

[18] Gruen RL Elliott JH Nolan ML. Sustainability science: an integrated approach for health-programme planning. Lancet. 2008;372(9649):1579–1589. 10.1016/S0140-6736(08)61659-1. 18984192

[19] African Programme for Onchocerciasis Control (APOC); World Health Organization (WHO). Guidelines for conducting an evaluation of the sustainability of CDTI projects. APOC and WHO; 2004. https://www.who.int/apoc/publications/guidelinesevalsustainabilitycorrectedversionsept04.pdf. Accessed April 10, 2019.

[20] Chilundo BGM Cliff JL Mariano ARE Rodríguez DC George A. Relaunch of the official community health worker programme in Mozambique: is there a sustainable basis for iCCM policy? Health Policy Plan. 2015;30(suppl 2):ii54–ii64. 10.1093/heapol/czv014. 26516151 PMC4625760

[21] Sarriot EG Winch PJ Ryan LJ. Qualitative research to make practical sense of sustainability in primary health care projects implemented by non-governmental organizations. Int J Health Plann Manage. 2004;19(1):3–22. 10.1002/hpm.743. 15061287

[22] Shediac-Rizkallah MC Bone LR. Planning for the sustainability of community-based health programs: conceptual frameworks and future directions for research, practice and policy. Health Educ Res. 1998;13(1):87–108. 10.1093/her/13.1.87. 10178339

[23] Wiltsey Stirman S Kimberly J Cook N Calloway A Castro F Charns M. The sustainability of new programs and innovations: a review of the empirical literature and recommendations for future research. Implement Sci. 2012;7(1):17. 10.1186/1748-5908-7-17. 22417162 PMC3317864

[24] Schell SF Luke DA Schooley MW. Public health program capacity for sustainability: a new framework. Implement Sci. 2013;8(1):15. 10.1186/1748-5908-8-15. 23375082 PMC3599102

[25] Yin RJ. Case Study Research: Design and Methods. 4th ed. Thousand Oaks, CA: Sage; 2009.

[26] Lifson AR Demisse W Ketema K. Failure to test for HIV in rural Ethiopia: knowledge and belief correlates and implications for universal test and treat strategies. J Int Assoc Provid AIDS Care. 2013;12(5):306–311. 10.1177/2325957413488199. 23744773

[27] Fauci AS. No more excuses. We have the tools to end the HIV/AIDS pandemic. *The Washington Post*. January 8, 2016. https://www.washingtonpost.com/opinions/no-more-excuses-we-have-the-tools-to-end-the-hivaids-pandemic/2016/01/08/a01cc876-b611-11e5-a842-0feb51d1d124_story.html. Accessed April 28, 2019.

[28] Reynolds LJ Camlin CS Ware NC Seeley J. Exploring critical questions for the implementation of “universal test and treat” approaches to HIV prevention and care. AIDS Care. 2016;28(suppl 3):1–6. 10.1080/09540121.2016.1178960. 27421046

[29] Maggard KR Hatwiinda S Harris JB. Screening for tuberculosis and testing for human immunodeficiency virus in Zambian prisons. Bull World Health Organ. 2015;93(2):93–101. 10.2471/BLT.14.135285. 25883402 PMC4339958

[30] Mendelsohn S Aluda C Reinaldo O Shigayeva A Hilderbrand K Goemaere E. Implementation of HIV “test-and-treat” strategy in Malawi prisons; experience, challenges and effectiveness. Paper presented at: 11th International Workshop on HIV Treatment Pathogenesis and Prevention Research in Resource Limited Settings; May 2017; Lilongwe, Malawi. http://regist2.virology-education.com/Abstractbook/2017/11interest.pdf. Accessed April 28, 2019.

[31] Topp SM Sharma A Moonga CN Chileshe C Magwende G Henostroza G. Evaluation of a health system strengthening initiative in the Zambian prison system. BMJ Glob Health. 2018;3(1):e000614. 10.1136/bmjgh-2017-000614. 29564162 PMC5859816

[32] Sridhar D McKee M Ooms G. Universal health coverage and the right to health: from legal principle to post-2015 indicators. Int J Health Serv. 2015;45(3):495–506. 10.1177/0020731415584554. 26077857

[33] Todrys KW Amon JJ. Criminal justice reform as HIV and TB prevention in African prisons. PLoS Med. 2012;9(5):e1001215. 10.1371/journal.pmed.1001215. 22589705 PMC3348151

[34] Hushie M. Public-non-governmental organisation partnerships for health: an exploratory study with case studies from recent Ghanaian experience. BMC Public Health. 2016;16(1):963. 10.1186/s12889-016-3636-2. 27618964 PMC5020518

[35] Srivastava A Bhattacharyya S Gautham M Schellenberg J Avan BI. Linkages between public and non-government sectors in healthcare: a case study from Uttar Pradesh, India. Glob Public Health. 2016;11(10):1216–1230. 10.1080/17441692.2016.1144777. 26947898

[36] Topp SM Moonga CN Luo N. Exploring the drivers of health and healthcare access in Zambian prisons: a health systems approach. Health Policy Plan. 2016;31(9):1250–1261. 10.1093/heapol/czw059. 27220354 PMC5035781

[37] Topp SM Moonga CN Mudenda C. Health and healthcare access among Zambia's female prisoners: a health systems analysis. Int J Equity Health. 2016;15(1):157. 10.1186/s12939-016-0449-y. 27671534 PMC5037633

[38] Boyer S Iwuji C Gosset A.; ANRS 12249 TasP study group. Factors associated with antiretroviral treatment initiation amongst HIV-positive individuals linked to care within a universal test and treat programme: early findings of the ANRS 12249 TasP trial in rural South Africa. AIDS Care. 2016;28(suppl 3):39–51. 10.1080/09540121.2016.1164808. 27421051 PMC5096681

[39] Agboola C. Memories of the ‘inside’: conditions in South Africa women's prisons. SA Crime Quarterly. 2016;56:19–26. https://www.ajol.info/index.php/sacq/article/view/138588. Accessed May 8, 2019.

[40] Dissel A. “By the Grace of God”: Staffing Correctional Facilities. Cape Town, South Africa: Sonke Gender Justice; 2016. https://genderjustice.org.za/publication/by-the-grace-of-god/. Accessed April 28, 2019.

[41] Sifunda S Reddy P Braithwaite RL Stephens T Ruiter RAC van den Borne B. Psychosocial determinants of risky sexual behaviour amongst South African male prison inmates in KwaZulu-Natal and Mpumalanga Provinces. Int J Prison Health. 2012;8(3/4):151–162. 10.1108/17449201211285021. 25758149

[42] Bond V Hoddinott G Viljoen L Simuyaba M Musheke M Seeley J. Good health and moral responsibility: key concepts underlying the interpretation of treatment as prevention in South Africa and Zambia before rolling out universal HIV testing and treatment. AIDS Patient Care STDS. 2016;30(9):425–434. 10.1089/apc.2016.0114. 27610464 PMC5035365

[43] Larmarange J Diallo MH McGrath N.; ANRS 12249 TasP Study Group. The impact of population dynamics on the population HIV care cascade: results from the ANRS 12249 Treatment as Prevention trial in rural KwaZulu-Natal (South Africa). J Int AIDS Soc. 2018;21(Suppl 4):e25128. 10.1002/jia2.25128. 30027600 PMC6053480

[44] Topp SM Mwamba C Sharma A. Rethinking retention: mapping interactions between multiple factors that influence long-term engagement in HIV care. PLoS One. 2018;13(3):e0193641. 10.1371/journal.pone.0193641. 29538443 PMC5851576

[45] Pilgrim N Musheke M Raymond HF. Quality of care and HIV service utilization among key populations in Zambia: a qualitative comparative analysis among female sex workers, men who have sex with men and people who use drugs. AIDS Care. 2019;31(4):460–464. 10.1080/09540121.2018.1524119. 30257574

